# Standing Genetic Diversity and Transmission Bottleneck Size Drive Adaptation in Bacteriophage Qβ

**DOI:** 10.3390/ijms23168876

**Published:** 2022-08-09

**Authors:** Pilar Somovilla, Alicia Rodríguez-Moreno, María Arribas, Susanna Manrubia, Ester Lázaro

**Affiliations:** 1 Centro de Astrobiología (CAB), CSIC-INTA, Ctra. de Torrejón Km 4, Torrejón de Ardoz, 28850 Madrid, Spain; 2 Centro Nacional de Biotecnología (CNB-CSIC), c/Darwin 3, 28049 Madrid, Spain; 3 Grupo Interdisciplinar de Sistemas Complejos (GISC), Madrid, Spain

**Keywords:** standing genetic diversity, de novo mutations, adaptation, transmission bottleneck size, RNA viruses, bacteriophage Qβ, molecular evolution

## Abstract

A critical issue to understanding how populations adapt to new selective pressures is the relative contribution of the initial standing genetic diversity versus that generated de novo. RNA viruses are an excellent model to study this question, as they form highly heterogeneous populations whose genetic diversity can be modulated by factors such as the number of generations, the size of population bottlenecks, or exposure to new environment conditions. In this work, we propagated at nonoptimal temperature (43 °C) two bacteriophage Qβ populations differing in their degree of heterogeneity. Deep sequencing analysis showed that, prior to the temperature change, the most heterogeneous population contained some low-frequency mutations that had previously been detected in the consensus sequences of other Qβ populations adapted to 43 °C. Evolved populations with origin in this ancestor reached similar growth rates, but the adaptive pathways depended on the frequency of these standing mutations and the transmission bottleneck size. In contrast, the growth rate achieved by populations with origin in the less heterogeneous ancestor did depend on the transmission bottleneck size. The conclusion is that viral diversification in a particular environment may lead to the emergence of mutants capable of accelerating adaptation when the environment changes.

## 1. Introduction

One of the most distinctive features of RNA virus replication is its high error rate in the order of 10^−3^ to 10^−5^ mutations per copied nucleotide [1,2]. This fact, together with short generation times and large populations, leads to the generation of highly heterogeneous populations that are commonly described as quasispecies, a term referring to the dynamic distribution of related genomes in which nontrivial interactions are established [3,4,5,6,7,8,9]. In this scenario, the consensus sequence (corresponding to the most represented nucleotide at each genomic position) provides little information on the population’s genetic diversity, and the use of methodologies such as deep sequencing [10,11,12] or molecular and/or biological cloning [13,14,15] becomes necessary to obtain this information.

Mutant spectra are not static entities. In addition to the unavoidable errors associated to replication, they can be modified by factors such as population bottlenecks [16,17,18] or environmental changes [19,20]. Both disturbances reduce the diversity of the mutant spectrum due to the random sampling of subsets of genomes (in the case of population bottlenecks) or the fitness advantages of particular mutants over others (in the case of new selective pressures). Subsequent replication rounds regenerate the diversity of the quasispecies, although during the process, the mutant spectrum can undergo profound changes in its structure [21].

The relevance of population bottlenecks for viral adaptation was extensively studied in previous evolution experiments where the number of viruses that initiated a new infection varied [22,23,24]. When this number is low, competition among genomes is minimised, and evolution is governed by genetic drift [16,18,25]. Plaque-to-plaque transfers in which the viral progeny contained in a single lytic plaque is serially transferred represent an extreme example of population bottlenecks in which, due to the extremely low multiplicity of the used infection, the population size at each transfer is reduced to a single individual (the one that originates the lytic plaque). Viral transmission under this regime fixes mutations regardless of their selective value, usually leading to initial fitness losses that eventually stabilise around a fitness stationary state with fluctuations that were attributed to the fixation of compensatory mutations [26,27,28,29]. In contrast, viral transmission through large populations maintains population genetic diversity, thus favouring the action of natural selection and increasing population fitness [23,30,31].

In the current study, we focused on understanding the relevance for the viral adaptation of pre-existent genetic variation versus that generated de novo in bacteriophage Qβ [32,33], an RNA virus of positive polarity with a genome of 4217 nucleotides that infects *Escherichia coli* through binding to the conjugative pilus. Its optimal replication temperature in the laboratory is 37 °C and, as other RNA viruses, it replicates with high error rate, estimated in 1.4 × 10^−4^ errors per copied nucleotide [34]. The quasispecies structure was first described in Qβ and has since been a broadly used model for the experimental evolution of RNA viruses [15,35,36,37,38,39,40,41]. The motivation for this research was a previous experiment [42] in which we compared the dynamics of adaptation to 43 °C of two Qβ populations, denoted Qβ-t2 and Qβ-t25. These populations originated from the same biological clone, but differed in the number of transfers (2 or 25, respectively) that they had previously experienced at optimal temperature (37 °C). Population Qβ-t25 increased its growth rate at 43 °C, much faster than population Qβ-t2, which was accompanied by the rapid fixation of mutations and the loss of the only two polymorphisms (A2187C and C3065U) observed in the consensus sequence before the temperature change [42]. The loss of A2187C was intriguing, since this mutation had been observed in many evolutionary lineages propagated in our lab at both 37 and 43 °C [37,38], including those obtained from Qβ-t2 evolved at 43 °C [42]. Our interpretation was that the propagation of Qβ for 25 transfers at 37 °C expanded the mutant spectrum, which, as a consequence, included minority genomes with beneficial effects at 43 °C that would have been rapidly selected upon exposure to this condition, displacing those genomes containing mutation A2187C. The fact that all transfers were carried out through large transmission bottlenecks (about 10^7^ pfu at each transfer) prevented the loss of minority genomes through genetic drift. 

To test this idea, we explored the relationships among genetic heterogeneity, the size of the transmission bottleneck, and the magnitude of the fitness change experienced by different populations of Qβ. As reported in this contribution, the results show that transmission bottleneck size influences the adaptive pathways followed during the propagation of Qβ-t25 at 43 °C, but does not affect the magnitude of the increase in growth rate. In contrast to this, the adaptation of a biological clone of Qβ to 43 °C requires a population bottleneck size above a minimal value. We conclude that the effect of the transmission bottleneck size on viral adaptation is strongly dependent on the pre-existent genetic diversity of the ancestral population. 

## 2. Results

### 2.1. Replication Increases the Genetic Diversity of Qβ Populations and Gives Rise to a Mutant Spectrum Containing Mutations Relevant for Adaptation to 43 °C

**Figure 1:**
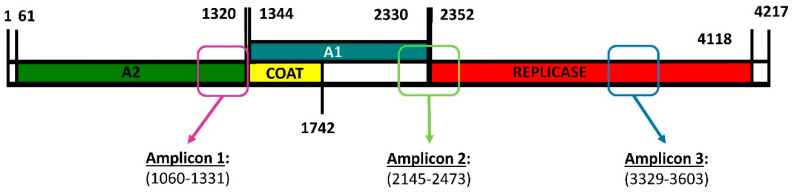
Genome map of bacteriophage Qβ showing the encoded proteins and the regions subjected to deep sequencing. A2 is the maturation protein involved in the internalisation of the viral genome and the exit of the mature viral particles. The coat protein is the major capsid protein that can sometimes be extended, giving rise to a minor capsid protein denoted A1. The replicase is the protein that copies the RNA genome. Primers used to generate the amplicons shown in the figure are described in Section 4.

To estimate whether Qβ replication under standard conditions (37 °C) increased the genetic diversity in the viral population, we carried out a deep sequencing analysis of three amplicons (Figure 1) obtained for populations Qβ-t2 and Qβ-t25, which differed in the number of transfers (2 or 25, respectively) that they had experienced [42]. We chose these three regions to be amplified because, on the basis of our previous experience, they contain some of the nucleotides that most frequently undergo mutations during Qβ adaptation at 43 °C.

We carried out a bioinformatics processing of the total number of paired reads obtained per amplicon (see Section 4). We also performed a control experiment to determine the threshold for the minimal frequency that haplotypes must reach to be included in our studies (Figure S1 and Table S1). On the basis of the obtained results, a threshold of 0.05% seemed adequate for our analyses. 

The consensus sequences of all amplicons coincided with that of the wild-type virus (Qβ_wt_, see Section 4) with the only exception of amplicon 2 in Qβ-t25, where the mutation A2187C was clearly observable.

Four different measures (defined in Section 4) were used to estimate the genetic diversity found in the amplicons: haplotypic density (*HD*), maximal mutation frequency (*Mf*), Shannon entropy normalised by the number of haplotypes (*H_S_*), and nucleotide diversity (*π*). All of them are frequently used to estimate the diversity contained in viral quasispecies [43,44]. The values for the assayed diversity indices were always higher for the amplicons corresponding to Qβ-t25 (Table 1). The representation of the frequency of the different haplotypes as a function of their Hamming distance or their rank (Figures S2 and S3) showed that, under the used conditions and for the number of analysed transfers, the genetic diversity contained in Qβ populations increases with the number of generations experienced from a clonal origin.

We also identified the mutations that were represented above 0.5% in at least one of the three Qβ-t25 samples (Figure S1 and Table 2) or in Qβ-t2. We found a total of 19 mutations that were above the threshold in Qβ-t25, with the majority being present in all samples (Table 2). This number decreased to six mutations in population Qβ-t2. 

More than half of the mutations represented above 0.5% (11 out of 19) in Qβ-t25 had been detected in the consensus sequence of this or other Qβ populations propagated at 43 °C [15,36,37,38,40,42]. The number of mutations that had been previously identified during Qβ adaptation to 43 °C increased to five out of the six mutations represented above 2%, and to all of those represented above 5% (see Table 2). These results show that, during propagation at 37 °C, the mutant spectrum of Qβ can become enriched in mutations useful for adaptation to higher temperatures.

### 2.2. Loss of Mutation A2187C upon Propagation of Qβ-t25 to 43 °C Is Not Due to a Negative Effect 

In our previous study, we observed that mutation A2187C was lost in all replicate lineages corresponding to the propagation of Qβ-t25 at 43 °C with large transmission bottlenecks (10^7^ pfu at each transfer) [42]. The loss was striking, since that same mutation could be identified upon propagation of Qβ-t2 at 43 °C, and also in previous experiments carried out at both 37 and 43 °C [37,38], suggesting that change A2187C could have a general beneficial effect or be prone to be hitchhiked by other advantageous mutations.

The site-directed mutagenesis of expression vector pBRT7Qβ [45,46] allowed for us to generate a single mutant containing A2187C (virus Qβ_A2187C_). The growth rate of Qβ_A2187C_ at 37 °C (16.7 ± 1.7 doublings per two hours) did not differ significantly (*p* = 0.382; Mann–Whitney test) from that of a wild-type virus obtained upon expression of nonmutagenised pBRT7Qβ (Qβ_wt_; see Section 4) (16.0 ± 1.0 doublings per two hours). In contrast, at 43 °C, virus Qβ_A2187C_ had a growth rate (7.6 ± 0.6 doublings per two hours) that was slightly higher than that of Qβ_wt_ (6.4 ± 0.8 doublings per two hours) (*p* = 0.029; Mann–Whitney test), indicating that the loss of A2187C was not due to a negative effect on Qβ replication at 43 °C. 

A possible explanation for the loss of A2187C could be that, in the mutant spectrum of Qβ-t25, this mutation was combined with others, resulting in a harmful effect for replication at 43 °C. To test this idea, we isolated six biological clones from population Qβ-t25, and determined their consensus sequences and their growth rates at 43 °C. The only preferential association among mutations that could be detected was A2187C with C3065U (the other mutation that was present in the consensus sequence of Qβ-t25). This link between both mutations provides a circumstantial explanation for their simultaneous loss upon adaptation to 43 °C. Growth rate values showed no significant differences that could be attributed to the presence or absence of A2187C (*p* = 0.35, Kruskal–Wallis test) (Table 3).

An alternative explanation for the loss of A2187C could be that population Qβ-t25 contained some minority variants whose effects at 43 °C were more advantageous than those of the genomes containing A2187C. If that were true, the selection of those minority mutants would depend on their frequency in the mutant spectrum and on the bottleneck size used at each transfer during adaptation at 43 °C. Small transmission bottlenecks could cause the loss of the low-frequency variants as a result of stochasticity through the bottleneck and the fixation of some neutral mutations due to genetic drift, while larger bottlenecks would be compatible with their persistence and increased frequency throughout the transfer series.

### 2.3. Population Size Influences the Adaptive Pathways Followed during Propagation of Qβ-t25 at 43 °C, but Does Not Affect the Magnitude of the Increase in Growth Rate

**Figure 2:**
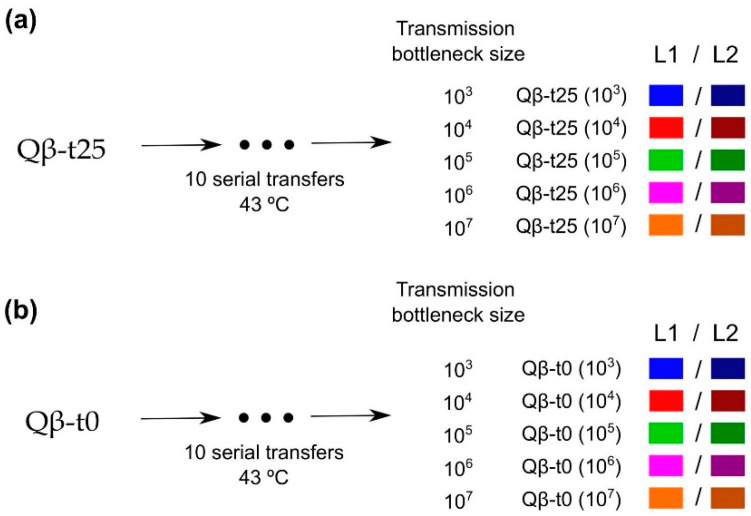
Scheme of the evolution experiment. Populations (**a**) Qβ-t25 and (**b**) Qβ-t0 were propagated at 43 °C through 10 serial transfers each (see Section 4.4) that were carried out using different transmission bottleneck sizes. Evolutionary lineages obtained at Transfer 10 are denoted with the name of the ancestor followed by the transmission bottleneck size used at each transfer. L1 and L2 were used to distinguish the two replicas of each condition. Colour code is maintained all through this paper.

To analyse the impact of the transmission bottleneck size on adaptation to 43 °C, population Qβ-t25 and a clonal population (Qβ-t0; see Section 4) were propagated through 10 serial transfers with the number of pfu that initiated infection at each transfer, at the levels of 10^3^, 10^4^, 10^5^, 10^6^, or 10^7^ pfu (5 levels × 2 replicates = 10 evolutionary lineages for each initial phage population; Figure 2). Previous experiments carried out in our group showed that Qβ replication at 37 °C does not increase the viral growth rate at 43 °C [42].

As observed in a previous experiment focused on Qβ adaptation to 43 °C [42], mutation A2187C was always selected against regardless of the population size used for viral transmission. In contrast to this, the propagation of Qβ-t25 for 35 additional transfers at 37 °C gave rise to a population that kept in its consensus sequence the two polymorphic mutations that were already present in the ancestor (A2187C and C3065U), in addition to others (U574C, U1295C, U1295G, A1930G, C2201U, G2741A, and G3945A).

**Figure 3:**
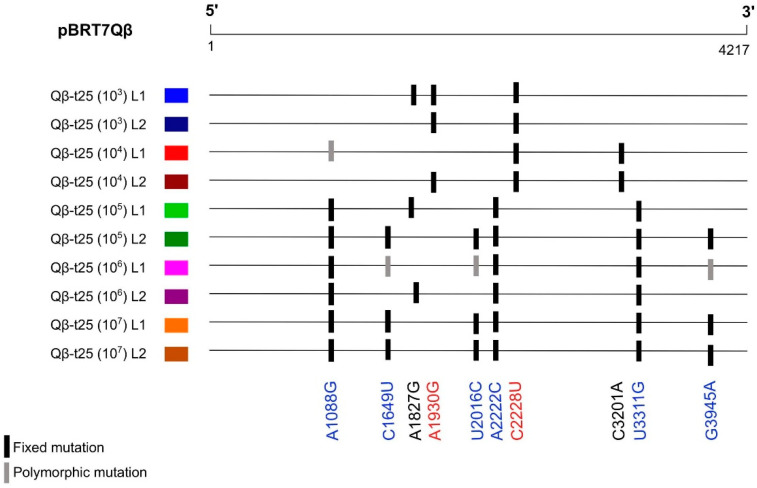
Mutations present in the consensus sequences of two or more of evolutionary lineages propagated at 43 °C from Qβ-t25 (see Figure 2a). A complete list of all the mutations in these populations is shown in Table S2. Mutations in red correspond to those more represented in the evolutionary lineages propagated at small transmission bottlenecks (10^3^ and 10^4^ pfu), whereas those in blue were the most frequent when the transmission bottleneck was larger (≥10^5^ pfu).

Focusing on the mutations that were observed in the consensus sequences corresponding to the whole genome of at least two of the evolutionary lineages (Table S2), we observed that adaptation through small transmission bottlenecks (10^3^ and 10^4^ pfu) took place preferably through mutations A1930G and C2228U, whereas population sizes above 10^5^ pfu involved A1088G, C1649U, U2016C, A2222C, U3311G, and G3945A (Figure 3). The latter mutations were also identified in the evolutionary lineages obtained by Somovilla et al. [42] upon the propagation of Qβ-t25 at 43 °C using a population size of 10^7^ pfu. 

The location of some of these mutations (A1088G, A2222C, and C2228U) in the genomic regions analysed through deep sequencing allowed for us to make some interesting observations. According to the results shown in Table 2, mutation C2228U was present in Qβ-t25 with a frequency of 8.5 ± 0.1%, higher than that of A1088G (0.53 ± 0.03%) or A2222C (0.38 ± 0.15). This means that genomes containing C2228U had higher probabilities to be imposed at small transmission bottlenecks. In contrast to this, an increase in the representation of genomes containing A1088G and/or A2222C, which were present at lower frequencies, would require larger populations in order to not be lost due to stochastic effects. Thus, it seems that the adaptive pathways to 43 °C followed by a heterogeneous population are determined by the interplay between the population size and the pre-existent genetic diversity. 

**Figure 4:**
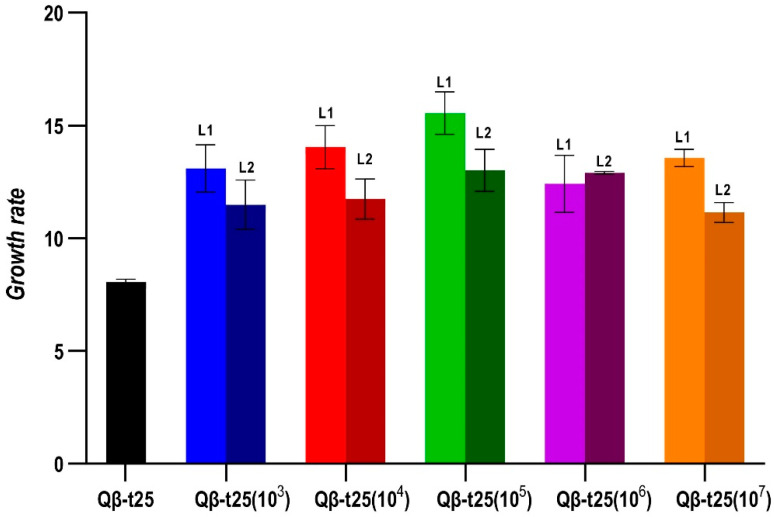
Growth rate values at 43 °C obtained for the evolutionary lineages propagated from Qβ-t25. The value obtained for the ancestor is also shown. Each bar represents the average of three replicas, and the error bars correspond to the standard deviation.

The determination of the growth rates at 43 °C of all evolutionary lineages obtained at Transfer 10 from Qβ-t25 (Figure 4) showed that all of them had significantly increased their values with respect to the ancestor (*p* < 0.05 for all pairwise comparisons between the growth rate of Qβ-t25 and each evolutionary lineage, Mann-Whitney test), although there were no significant differences among lineages that could be attributed to the population size (*p* = 0.21, Kruskal–Wallis test).

To find out whether the advantage provided by the mutations fixed in the consensus sequences of the evolved lineages was due to a direct beneficial effect at 43 °C or to their association with others, we determined the growth rate (at both 37 and 43 °C) of several single mutants generated by site-directed mutagenesis (Table 4). The chosen mutations were A1088G, A1930G, C2228U, and U3311G. G1088A and C2228U were found at frequencies above 0.5% in population Qβ-t25 (0.53 ± 0.03% and 8.5 ± 0.1%; Table 2). That difference in their abundance possibly determined their different fate (loss of or increase in frequency) in the evolutionary lineages as a function of the bottleneck transmission size (Figure 3). Mutations A1930G and U3311G, which were located outside the regions analysed by deep sequencing, were also fixed in some of the evolved populations (Figure 3).

There were no significant differences between the growth rates of the different mutants and that of virus Qβ_wt_ at either 37 or 43 °C (*p* > 0.05 for all pairwise comparisons at a given temperature between the mutants and virus Qβ_wt_, Mann–Whitney test). Thus, none of the tested mutations provided an increase in viral growth rate when it was present as a single mutation in the genome. This agrees with our previous observation that the final fate of particular mutations at 43 °C depends on their mutational context [15]. 

### 2.4. Adaptation to 43 °C of a Biological Clone of Qβ Requires a Population Bottleneck Size above a Minimal Value

**Figure 5:**
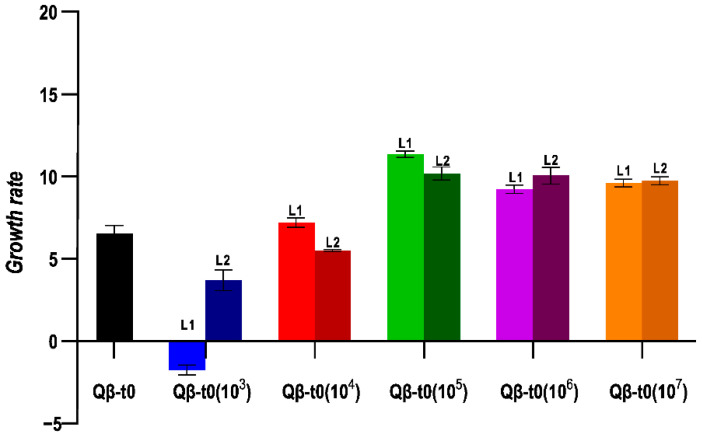
Growth rate values at 43 °C obtained for the evolutionary lineages propagated from Qβ-t0. The value obtained for the ancestor is also shown. Each bar represents the average of three replicas, and the error bars correspond to the standard deviation.

To analyse whether the transmission bottleneck size influences evolution differently depending on the heterogeneity of the ancestral population, we also carried out the same evolution experiment using as ancestor a population that had only replicated for the necessary number of generations to generate a lysis plaque, Qβ-t0 (Figure 2b). In this case, the growth rate values obtained at Transfer 10 showed significant differences depending on the population size used for viral transmission (Figure 5). Propagation through small populations (10^3^ in L1, and L2 and 10^4^ in L2) produced significant decreases in growth rate (*p* < 0.05 for all pairwise comparisons between the evolutionary lineages indicated and virus Qβ-t0, Mann–Whitney test). On the other hand, the propagation of Qβ-t0 through population sizes ≥ 10^5^ was associated to significant increases in growth rate at 43 °C (*p* < 0.05 for all pairwise comparisons between the evolved lineages and virus Qβ-t0, Mann–Whitney test). In good agreement with these observations, the results of the statistical tests show that there was a significant influence of the population size on the values of the growth rate at 43 °C reached after 10 transfers (*p* < 0.001, Kruskal–Wallis test).

## 3. Discussion

A relevant question in evolutionary biology refers to the relative importance in adaptation of the de novo generated diversity versus the standing diversity of populations [47,48,49,50,51,52,53,54,55,56,57]. The standing genetic diversity acquires greater significance in the case of RNA virus populations, in which the most frequent variant is always surrounded by a cloud of mutants, some of which could become beneficial when there is a change in the environmental conditions. There are studies showing the existence of minority genomes in viral quasispecies with selective advantages in previous environments experienced by the population, which speeds up adaptation when the population faces similar circumstances [58,59,60]. However, the influence on adaptation of the progressive expansion and diversification of the mutant spectrum that occurs when a virus evolves under constant conditions in the absence of apparent selective pressures is more challenging to determine [19,21,61].

In the current study, population Qβ-t25, which always replicated at 37 °C, contained a significant fraction of mutations represented above 0.5%. These mutations had previously been detected in the consensus sequence of Qβ populations propagated at 43 °C (Table 2) [15,36,37,38,40,42]. Some of them (U1295C and A2187C) had also been identified in adaptation experiments carried out at 37 °C, but the remaining ones had never been observed at that temperature. Taken together, the results show that the propagation of Qβ under standard conditions causes a diversification of the mutant spectrum, favouring the generation of particular mutations that may facilitate adaptation to selective pressures that the virus had not previously experienced. 

Recent studies carried out with the hepatitis C virus propagated under constant conditions in cell cultures showed a similar diversification of the mutant spectrum, which included some variants that were resistant to particular antiviral drugs to which the virus had not been exposed [21,62,63]. These results, like ours, show how the process of viral replication in a constant environment may lead to the emergence of mutants with selective advantages under new conditions. The spread of molecular populations on the space of neutral or quasineutral sequences is unavoidable and has been documented in multiple viruses, playing an essential role in the adaptation of heterogeneous populations [64,65]. The longer the time during which a viral population has evolved in a constant environment is, the larger the fraction of rare mutants that it incorporates, and the broader the region of sequence space explored are. 

The different frequencies at which the mutations with potential beneficial effects at 43 °C were found in Qβ-t25 suggest that the population size used to initiate each transfer during the subsequent propagation of the virus at 43 °C could determine the adaptive pathways and probably the fitness values achieved. According to our expectations, and considering the mutations included in the amplicons sequenced through deep sequencing, propagation through small populations lead to the selection of mutation C2228U that was represented with higher frequency than that of A1088G or A2222C, which required larger populations to be present in the consensus sequences of the adapted populations. Despite observed differences in the followed adaptive pathways, there were no significant deviations in the growth rates that could be attributed to the population size at which the populations had been propagated. A possible explanation for this similarity in growth rates may lie in the de novo mutations acquired during the 10 transfers.

The fact that the consensus sequences of populations propagated through large transmission bottlenecks lacked mutation C2228U suggests that genomes containing A1088G and/or A2222C have higher fitness than those containing C2228U. However, we actually ignored the mutational context in which each of these mutations was present. The sequence of six biological clones isolated from population Qβ-t25 (Table 3) showed the presence of mutation A1930G in two of them, which fixed in the consensus sequence of populations propagated through small populations. However, it was not associated with C2228U, as it did after 10 passages at 43 °C. 

In contrast with the results described above, the propagation of a clonal population, also for 10 transfers at 43 °C, gave rise to evolutionary lineages that showed large differences in their growth rate values as a function of the transmission bottleneck size. Transmissions of 10^3^ or 10^4^ pfu at each transfer were incompatible with adaptation, perhaps due to the stochastic loss of beneficial mutations. An alternative explanation is that the generation of beneficial mutations at 43 °C is difficult when the number of viruses that replicate is not sufficiently large. The propagation of the same clonal population through population sizes ≥ 10^5^ pfu produced significant increases in growth rate, which is compatible with both explanations.

Propagation through small populations can also lead to the fixation of or increase in the frequency of some neutral mutations due to genetic drift or hitchhiking with beneficial mutations. Thus, some of the mutations shown in Table S2 could be irrelevant for adaptation to 43 °C and contingent to this particular experiment. However, mutations shown in Figure 3, which are coincident in several evolutionary lineages, are very likely to have a selective value at 43 °C and have increased their frequency for that reason.

The findings shown in this work illustrate the advantages for the adaptation of the pre-existent genetic diversity in RNA viruses. Previous results of our group also showed that Qβ populations that were slightly mutagenised using low concentrations of 5-azacytidine rapidly fixed a set of mutations upon propagation at increased temperature [38]. Most of these mutations differed from those fixed when the ancestral population had not been subjected to mutagenesis, suggesting that the adaptive process took place through standing genetic diversity, which was different from that contained in nonmutagenised populations [66]. 

Although we did not compare the sequences obtained from Qβ-t25 and Qβ-t0 in this work, the results of a previous study comparing the sequences obtained from Qβ-t25 and Qβ-t2 after propagation at 43 °C for 60 transfers at large populations showed a higher coincidence of mutations in the lineages that had evolved from Qβ-t25 [42]. Our reasoning was that, in a more heterogeneous population, standing diversity suffices to trigger adaptation, while in a less heterogeneous population, it must resort to newly generated mutations.

The determination of the growth rate values of the mutants containing single mutations, chosen among those present at low frequencies in Qβ-t25 and/or present in the consensus sequences of the lineages evolved at 43 °C, showed that they had nonsignificant effects at 37 and 43 °C. This finding suggests that the fitness advantage they provide depends on the presence of additional mutations whose combination is advantageous at 43 °C. Do these combinations already exist in the ancestral population as minority genomes or are they generated during propagation at 43 °C? The answer is unknown, but if the latter possibility is the right one, the most probable explanation for the differences in the adaptation of populations Qβ-t0 and Qβ-t25 would be that the presence of particular mutations in the more heterogeneous ancestor provides a genomic context that favours the acquisition of new mutations beneficial at 43 °C. This possibility is in good agreement with other studies showing that neutral or deleterious mutations may provide access to adaptive pathways that might otherwise be inaccessible [67,68,69]. The fact that many of the mutations present above 0.5% in Qβ-t25 were not detected in the consensus sequence when propagation at 37 °C had been extended for a longer number of transfers shows that they did not have a similar effect at this temperature. Taking this fact into account, at least a fraction of the mutations present at low frequency in Qβ-t25 could simply represent the most probable genetic changes that the wild virus can generate during propagation at 37 °C. The increase in their representation would depend on the subsequent selective pressures with which the viral population has to deal. Further experiments are in progress to determine the role of standing genetic diversity under selective pressures different from temperature increases. 

## 4. Materials and Methods

### 4.1. Viral Populations, Bacteria, and Standard Procedures for Infection

Plasmid pBRT7Qβ, which contains the cDNA of bacteriophage Qβ cloned in the plasmid pBR322 [45,46], was a generous gift from Professor CK Biebricher (Max Planck Institute for Biophysical Chemistry). It was used to transform *E. coli* DH5-α, which can express the phage, but cannot be infected since it lacks the F pilus. The supernatant of an overnight culture obtained from a transformed colony was used to infect *E. coli* Hfr (Hayes) in semisolid agar at a multiplicity of infection (moi) that allowed for the generation of well-separated lytic plaques. Under these conditions, there is a high probability that each lytic plaque results from the replication of a single virus for a limited number of generations; thus, they can be considered to be biological clones. The viral progeny contained in several randomly chosen lytic plaques was sequenced and, in case there were no mutations relative to the cDNA cloned in pBRT7Qβ, it was considered to be analogous to the wild-type virus (Qβ_wt_). 

Standard infections in liquid medium were carried out in NB medium (8 g/L Nutrient Broth from Merck (Darmstadt, Germany) and 5 g/L NaCl) using fresh log-phase *E. coli* Hfr cultures with an OD_600_ between 0.6 and 0.8. After 2 h of incubation at 37 °C with good aeration (250 rpm) in a New Brunswick Scientific Innova 42 Incubator Shaker (Eppendorf, Enfield, CT, USA), cultures were treated with 1/20 volume of chloroform for 15 min at 37 °C with shaking (600 rpm). Viral supernatants were harvested upon centrifugation at 13,000× *g*, and viral titres determined by plaque assay. 

Population Qβ-t0 corresponded to the viral progeny contained in a lytic plaque obtained upon expression of pBRT7Qβ. As the virus Qβ_wt_ described above, it had no mutations with respect to the Qβ cDNA cloned in pBRT7Qβ. Populations Qβ-t2 and Qβ-t25 were previously described [42]. Briefly, a biological clone isolated upon expression of pBRT7Qβ was propagated through serial transfers in *E. coli* Hfr. Supernatants obtained at Transfers 2 and 25 corresponded to populations Qβ-t2 and Qβ-t25, respectively.

### 4.2. Isolation of Biological Clones

Biological clones corresponded to lytic plaques obtained in semisolid agar in *E. coli* Hfr grown at 37 °C. They were isolated by punching and removing the top and the bottom agar around well-separated lytic plaques as previously described [39,70,71]. The agar containing each plaque was transferred into a tube with 1 mL of phage buffer (1 g/L gelatine, 0.05 M Tris-HCl, pH 7.5, and 0.01 M MgCl_2_) and 50 μL of chloroform, and incubated for 1 h at 25 °C with shaking (900 rpm). After centrifugation at 13,000× *g* for 15 min to clarify the supernatant, the latter was stored over 25 μL of chloroform.

### 4.3. Site-Directed Mutagenesis

Plasmid pBRT7Qβ was used to engineer the single-mutant virus (Qβ_A2187C_, Qβ_G1088A_, Qβ_A1930G_, Qβ_C2228U_, and Qβ_U3311G_) containing the indicated mutations. Mutagenesis was carried out using the QuikChange Lightning Site-Directed Mutagenesis Kit (Agilent Technologies) and complementary primers containing the mutation to introduce in the viral genome (Table S3). The procedures to build and isolate the site-directed mutants were the same as those previously described for other Qβ mutants [39]. Lytic plaques generated in *E. coli* Hfr by viruses Qβ_A2187C_, Qβ_G1088A_, Qβ_A1930G_, Qβ_C2228U_, and Qβ_U3311G_ were picked and sequenced to test the presence of the desired mutation and the absence of any other that might have arisen during the mutagenetic process.

### 4.4. Evolution Experiment

Population Qβ-t25 was the ancestor of five replicate evolutionary lineages that differed in the number of pfu that was used to initiate the infection (10^3^, 10^4^, 10^5^, 10^6^, or 10^7^ pfu) (Figure 2a). After 2 h of replication at 43 °C under the same conditions as those described in Section 4.1, viral supernatants were harvested, and a fraction of each was used to initiate a new culture (a new transfer) using the same number of pfu as that in the initial infection. All lineages were propagated through 10 serial transfers at 43 °C. At each transfer, bacteria were freshly prepared by growing *E. coli* until an OD_600_ between 0.6 and 0.8. Evolutionary lineages were denoted as Qβ-t25(10^3^), Qβ-t25(10^4^), Qβ-t25(10^5^), Qβ-t25(10^6^), and Qβ-t25(10^7^) to indicate the ancestral population and the transmission bottleneck size used at each transfer. When necessary, numbers 1 and 2 were used to distinguish the two replicas of each condition. A parallel experiment (Figure 2b) was carried out using as ancestor, the biological clone Qβ-t0 described above (Section 4.1). In this case, the evolutionary lineages obtained at Transfer 10 were denoted as Qβ-t0(10^3^), Qβ-t0(10^4^), Qβ-t0(10^5^), Qβ-t0(10^6^), and Qβ-t0(10^7^).

A negative control in which undiluted bacteria were incubated in a culture medium in the absence of the virus was propagated following the same protocol as that for the experimental samples and plated at each transfer. When lytic plaques appeared, the corresponding transfer was discarded and repeated.

### 4.5. Growth Rate Determinations

Triplicate liquid cultures containing 10^8^ bacteria growing in exponential phase were inoculated with 10^4^ pfu in a final volume of 1 mL. After 2 h of incubation at 43 °C, the viral supernatants were collected as described above and titrated to estimate the growth rate, which was used as a surrogate of fitness. Previous assays carried out in our lab showed that bacteriophage Qβ grew exponentially under these conditions. Growth rates were expressed as the number of doublings per 2 h, and were calculated as log_2_(*N_f_*/*N*_0_), where *N*_0_ was the initial input of the virus, and *N_f_* the number of progeny pfu. A reference virus was always included in the assay. When *N_f_* was lower than *N*_0_, the resulting growth rate had a negative value.

### 4.6. RNA Extraction, cDNA Synthesis, PCR Amplification, and Nucleotide Sequencing

Viral RNA was prepared following standard procedures to determine the consensus sequence either from biological clones or from complex viral populations. RNAs were used for cDNA synthesis with the avian myeloblastosis virus reverse transcriptase (Promega), followed by PCR amplification using Expand High-Fidelity DNA Polymerase (Roche). The pairs of oligonucleotide primers used for RT-PCR were the following: P1 forward (5′CTTTAGGGGGTCACCTCACAC3′) with P1 reverse (5′GGATGGGTCACAAGAACCGT3′) to amplify from nucleotide position 10 to 1595, P2 forward (5′GACGTGACATCCGGCTCAAA3′^′^) with P2 reverse (5′CAACGGACGGAACATCTCCT3′^′^) to amplify from nucleotide position 1109 to 2787 and P3 forward (5′GTGCCATACCGTTTGACT3′) with P3 reverse (5′GATCCCCCTCTCACTCGT3′) to amplify from nucleotide position 2254 to 4195. PCR products were column-purified (Qiagen) and subjected to standard Sanger sequencing using Big Dye Chemistry (v3.1) with an automated sequencer (Abi 3730 XL, Applied Biosystems, Perkin Elmer). Sequences were assembled and aligned with Geneious Pro v4.8.5 (https://www.geneious.com, accessed on 15 February 2022). Mutations relative to the sequence of the Qβ cDNA present in plasmid pBRT7Qβ (virus Qβ_wt_) were identified using the same software. 

### 4.7. Next Generation Sequencing (NGS) of Populations Qβ-t2 and Qβ-t25

Viral RNA was extracted and purified from populations Qβ-t2 and Qβ-t25 using the Mini Kit Viral RNA QIAamp (Qiagen). It was amplified by RT-PCR using SuperScript II Reverse Transcriptase (Invitrogen) and Q5 Hot Star High fidelity (New England Biolabs) with three pairs of specific nucleotide primers that allowed to generate three amplicons (P1 forward: 5′GAATGTTGGTGACATACTTGCT3′ with P1 reverse: 5′TTGCCATGATCAAATTGACC3′ to amplify from nucleotide position 1037 to 1349, P2 forward: 5′GGTGCTTTCGGTAACATTGAG3′ with P2 reverse: 5′TGAATGAAATACACTCAGCCTCAG3′ to amplify from nucleotide position 2123 to 2516, and P3 forward 5′AGATTTCTTCTATGGGTAACGG3′ with P3 reverse 5′CCAGTATTAAATCGGCAGGAC3′ to amplify from nucleotide position 3306 to 3623) (Figure 1). PCR products were quantified and tested for quality (TapeStation 4200, Agilent Technologies), prior to Illumina Ultra Deep Sequencing analysis (MiSeq platform with 2 × 250 bp mode and v2 chemistry). The coverage per sample and amplicon was always higher than 200,000. The fastq files were processed using the software Galaxy (www.usegalaxy.org, accessed on 15 May 2021). First, the forward and reverse reads were paired [72] and subjected to quality analysis (Babraham Bioinformatics, FastQC; www.illumina.com, accessed on 15 May 2021). Q values were always >30. Alignment was carried out against the reference Qβ genome (the sequence of the Qβ cDNA present in the plasmid pBRT7Qβ), and mapped with the BWA algorithm [73,74,75]. The results were filtered by position and CIGAR chain. The total number of paired reads per amplicon was denoted *N_t_*. *N*_0_ refers to the number of paired reads obtained after bioinformatic processing. The different sequences or haplotypes contained in *N*_0_ (*H*_0_) were identified with the Collapse function of the Galaxy platform (www.usegalaxy.org, accessed on 30 June 2021).

To determine the threshold for the frequency of different haplotypes suitable for subsequent analysis, sample Qβ-t25 was amplified in two parallel RT-PCR that were sequenced in two different sequencing projects (Figure S1), which gave rise to three different ensembles of reads that were processed as described above. The percentage of coincident haplotypes between pair of samples, using three different thresholds (Table S1), gave us an estimation of whether the detected genetic changes corresponded to real mutations produced during viral replication or to methodological errors. The numbers of haplotypes and reads above the 0.05% threshold in *N*_0_ were denoted as *N* and *H*, respectively. The amplicons corresponding to population Qβ-t2 were obtained in the same sequencing project as that of Sample 1 of Qβ-t25.

Values obtained for parameters *N_t_*, *N*_0_, *H*_0_, *N*, and *H* are shown in Table S4. 

### 4.8. Determination of Diversity Indices

We used the following parameters as indicators of the diversity of the analysed viral populations, which were Qβ-t2 and Qβ-t25 (Sample 1; see Figure S1) [43,44]:

Haplotypic density (*HD*):

*HD* = *H*/*N* (1)

where *H* refers to the number of haplotypes in the ensemble of reads *N*.

Maximal mutation frequency (*Mf*):

(2) $$Mf=\sum \frac{p_{i}}{L}$$

where *p_i_* represents the frequency of each mutation in the ensemble of reads *N*. *L* is the length of each amplicon.

Shannon entropy normalised by the number of haplotypes (*H*):

(3) $$Hs=-\sum q_{i}log\left(q_{i}\right)/log\left(H\right)$$

where *q_i_* refers to the frequency of each haplotype in the ensemble of reads *N*.

Nucleotide diversity (*π*):

(4) $$\pi =\overset{L}{\underset{l=1}{\sum }}\frac{D_{l}}{L}$$

where *D_l_* corresponds to:

(5) $$D_{l}=\frac{N\left(N-1\right)-\sum_{i}n_{i}\left(n_{i}-1\right)}{N\left(N-1\right)}$$

and *n_i_* indicates the frequency of each of the four nucleotide possibilities at each genomic position (the sum runs from 1 to 4*L*).

Calculations were performed with an algorithm implemented in C.

## Figures and Tables

**Table 1:** Values of haplotypic density (*HD*), maximal mutation frequency (*Mf*), Shannon entropy normalised by the number of haplotypes (*H_S_*), and nucleotide diversity (*π*), obtained for the amplicons corresponding to Qβ-t2 and Qβ-t25.

Population	Amplicon ^2^	*HD* ^3^	*Mf* ^3^	*H_S_* ^3^	*π* ^3^
Qβ-t2	1	1.12	1.98	0.11	0.39
Qβ-t2	2	1.66	3.20	0.16	0.64
Qβ-t2	3	1.74	2.02	0.11	0.40
Qβ-t25 (Sample 1) ^1^	1	3.56	5.23	0.21	1.04
Qβ-t25 (Sample 1) ^1^	2	2.54	30.53	0.45	3.15
Qβ-t25 (Sample 1) ^1^	3	2.14	3.66	0.17	0.73

^1^ The origin of Sample 1 of population Qβ-t25 is described in Figure S1. ^2^ The location of each amplicon is shown in Figure 1. ^3^ The precise description of the parameters can be found in Section 4.8.

**Table 2:** Mutations represented above 0.5% in the deep sequencing analysis carried out with populations Qβ-t25 and Qβ-t2.

Mutation ^1^	Amplicon ^2^	Frequency (%)			
		Qβ-t25 Sample 1 ^3^	Qβ-t25 Sample 2 ^3^	Qβ-t25 Sample 3 ^3^	Qβ-t2
**A1088G**	1	0.52	0.51	0.57	<0.5
**U1295C**	1	1.35	1.31	1.32	<0.5
**U1295G**	1	0.52	0.14	0.13	0.67
**G1312A**	1	2.85	2.52	2.50	0.70
U1328G	1	0.99	0.67	0.54	<0.5
**A2187C**	2	68.80	68.88	69.80	1.00
C2189U	2	1.13	1.14	1.24	1.27
**C2201U**	2	6.21	5.94	5.75	<0.5
G2217A	2	2.23	2.14	1.88	<0.5
**A2222C**	2	0.22	0.52	0.44	<0.5
**G2223A**	2	2.93	2.85	2.92	<0.5
U2225C	2	1.69	1.76	1.73	<0.5
**C2228U**	2	8.62	8.60	8.35	<0.5
G2253A	2	1.11	1.06	0.98	<0.5
U2255C	2	0.87	0.83	0.76	<0.5
U2379G	2	0.19	0.87	0.95	<0.5
**U3402C**	3	0.90	0.79	1.00	<0.5
**C3545U**	3	1.29	0.77	0.96	0.67
A3603G	3	0.71	0.65	0.81	0.50

^1^ Mutations shown in bold had been previously detected during Qβ adaptation to increased temperature. ^2^ The location of each amplicon is shown in Figure 1. ^3^ The origin of Samples 1–3 of Qβ-t25 is shown in Figure S1 and described in Section 4.7.

**Table 3:** Growth rate values (43 °C) and mutations present in the consensus sequences of virus Qβ_wt_ and the biological clones isolated from population Qβ-t25.

Virus Clone	Mutations in the Consensus Sequence	Growth Rate ^1^
1	A2187C, C3065U, A3854G, A4148G	5.8 ± 1.4
2	G1650A, A1908G, A2187C, C3065U, C3254U, G3945A	1.1 ± 2.0
3	A1930G, G1820A, C1821U, A3470G,	4.7 ± 0.1
4	C723U, G2217A	6.4 ± 0.1
5	A1930G, G2223A	6.3 ± 0.6
6	A2187C, C2201U, C3065U,	7.3 ± 0.4
Qβ_wt_		6.0 ± 0.7

^1^ The values represent the average of three determinations ± the standard deviation.

**Table 4:** Growth rate values at 37 and 43 °C of virus Qβ_wt_ and several Qβ single mutants.

Viral Clone	Growth Rate at 37 °C ^1^	Growth Rate at 43 °C ^1^
Qβ_wt_	17.77 ± 0.35	5.95 ± 0.69
Qβ_A1088G_	17.07 ± 0.49	5.66 ± 0.81
Qβ_A1930G_	17.43 ± 0.73	6.91 ± 0.87
Qβ_C2228U_	17.77 ± 0.06	6.44 ± 0.72
Qβ_U3311C_	16.33 ± 0.38	6.75 ± 0.42

^1^ The values represent the average of three determinations ± the standard deviation.

## Data Availability

The datasets generated and/or analysed during the current study are available from the corresponding author on reasonable request.

## Supplementary Materials

The supporting information can be downloaded at: https://www.mdpi.com/article/10.3390/ijms23168876/s1.
