# Supplementary material for: Standing Genetic Diversity and Transmission Bottleneck Size Drive Adaptation in Bacteriophage Qβ
Source: Int J Mol Sci. 2022 Aug 9;23(16):8876. doi: 10.3390/ijms23168876 (PMC9408265; doi:10.3390/ijms23168876)
Supplement: Supplementary file 1 [file ijms-23-08876-s001.zip › Figure S1.pdf]

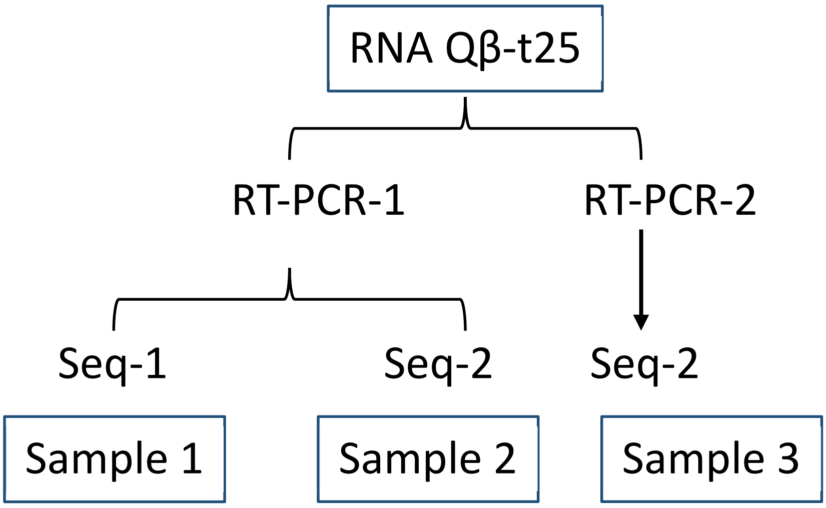

Figure S1. Scheme followed for deep sequencing analysis of population Qβ-t25. RNA obtained from population Qβ-t25 was amplified in two parallel RT-PCR, which were sequenced in two different sequencing projects (Seq-1 and Seq-2), rendering three different samples. The ensemble of paired reads obtained for each amplicon was subjected to bioinformatics processing (see section 4.7 of Materials and Methods). The percentage of coincident haplotypes in the samples (using three different threshold for the representation of haplotypes in the ensembles of paired reads obtained after bioinformatics analysis) was determined and used as an estimator of the reliability of our results (see Table S1).
