# Supplementary material for: Standing Genetic Diversity and Transmission Bottleneck Size Drive Adaptation in Bacteriophage Qβ
Source: Int J Mol Sci. 2022 Aug 9;23(16):8876. doi: 10.3390/ijms23168876 (PMC9408265; doi:10.3390/ijms23168876)
Supplement: Supplementary file 1 [file ijms-23-08876-s001.zip › Figure S2.pdf]

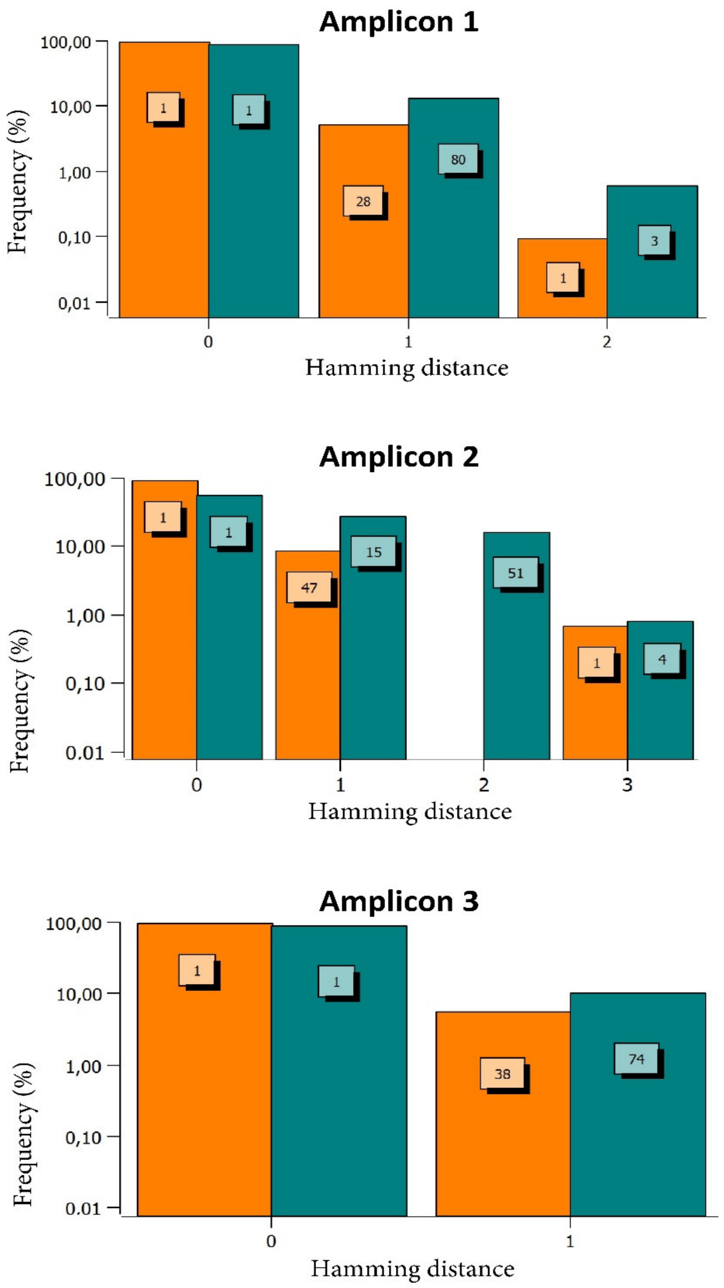

Figure S2. Representation of the frequency of haplotypes in the amplicons obtained for populations Qβ-t2 and Qβ-t25 as a function of the Hamming distance to the consensus sequence. Orange columns (population Qβ-t2), blue columns (sample 1 of population Qβ-t25; see Figure S1). The paired reads obtained for each amplicon were subjected to bioinformatics processing (see section 4.7 of Materials and Methods and Table S4). Haplotypes included in the analyses were those represented above the 0.05% threshold. Labels above the bars show the number of different haplotypes found at the indicated Hamming distance.
