# Supplementary material for: Standing Genetic Diversity and Transmission Bottleneck Size Drive Adaptation in Bacteriophage Qβ
Source: Int J Mol Sci. 2022 Aug 9;23(16):8876. doi: 10.3390/ijms23168876 (PMC9408265; doi:10.3390/ijms23168876)
Supplement: Supplementary file 1 [file ijms-23-08876-s001.zip › Figure S3.pdf]

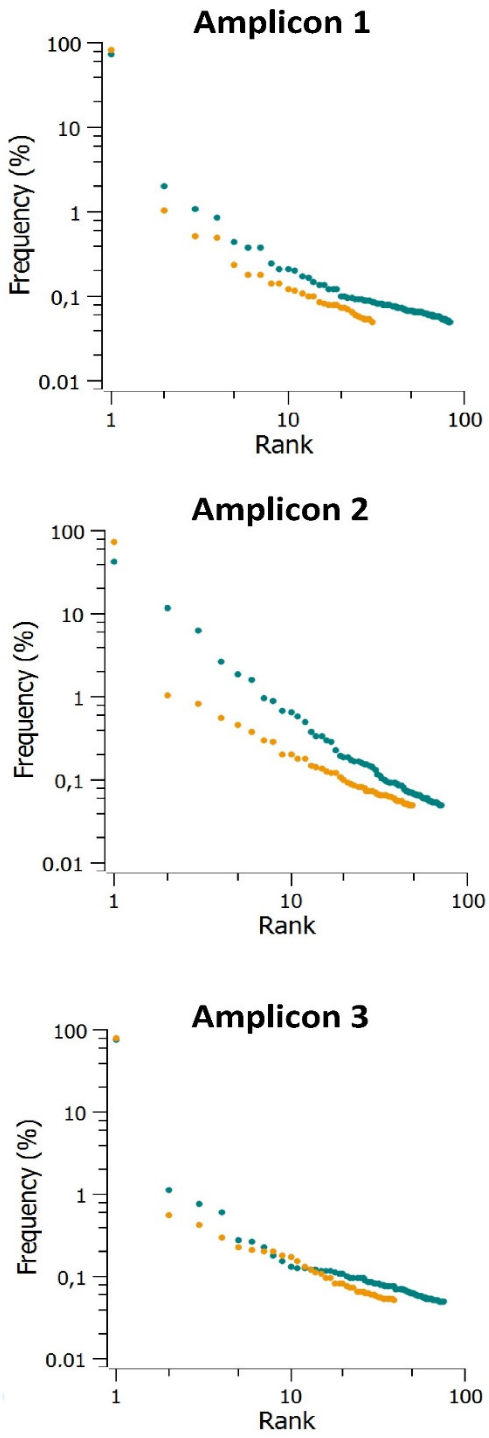

Figure S3. Distribution of the frequency of haplotypes in the amplicons obtained for populations Qβ-t2 and Qβ-t25 as a function of their rank. The paired reads obtained for each amplicon were subjected to bioinformatics processing (see section 4.7 of Materials and Methods and Table S4). Haplotypes included in the analysis were those represented above the 0.05% threshold. The rank indicates the number of order of each haplotype according to their representation in the population. Orange circles (population Qβ-t2), blue circles (sample 1 of population Qβ-t25; see Figure S1).
