# Supplementary material for: Standing Genetic Diversity and Transmission Bottleneck Size Drive Adaptation in Bacteriophage Qβ
Source: Int J Mol Sci. 2022 Aug 9;23(16):8876. doi: 10.3390/ijms23168876 (PMC9408265; doi:10.3390/ijms23168876)
Supplement: Supplementary file 1 [file ijms-23-08876-s001.zip › Table S1.pdf]

Table S1. Percentage of coincident haplotypes in the samples obtained in the deep sequencing analysis of population Qβ-t25.

Comparison[1]	Threshold 0.05 %[2]			Threshold 0.1 %[2]			Threshold 0.5 %[2]		
	Amp 1	Amp2	Amp 3	Amp1	Amp2	Amp3	Amp1	Amp2	Amp3
Sample 1 Sample 2	86.75 %	92.96%	90.67 %	95.24%	100.00%	80.00%	100.00%	100.00%	100.00 %
Sample 1 Sample 3	84.34 %	85.91%	82.67 %	85.71%	93.94%	70.00%	100.00%	100.00%	100.00 %
Sample 2 Sample 3	92.77 %	92.21%	90.00 %	95.83%	93.02%	74.07%	100.00%	100.00%	100.00 %

[1]Each cell shows the two samples that were compared (see Figure S1). The paired reads obtained for each of them were subjected to bioinformatics processing (see section 4.7 of Materials and Methods). [2]Each threshold indicates the minimum frequency that must be reached by a particular haplotype to be included in the analysis. Amp1, Amp2, and Amp3 refer to the three amplicons shown in Figure 1.
