# Supplementary material for: Standing Genetic Diversity and Transmission Bottleneck Size Drive Adaptation in Bacteriophage Qβ
Source: Int J Mol Sci. 2022 Aug 9;23(16):8876. doi: 10.3390/ijms23168876 (PMC9408265; doi:10.3390/ijms23168876)
Supplement: Supplementary file 1 [file ijms-23-08876-s001.zip › Table S2.pdf]

Table S2. Mutations present in the consensus sequences of the evolutionary lineages evolved at 43 °C from population Qβ-t25.

**Mutation**[1]	**Transmission bottleneck size (pfu)/Evolutionary lineage**									
	**10^3/ L1**	**10^3/ L2**	**10^4/L1**	**10^4/L2**	**10^5/L1**	**10^5/L2**	**10^6 /L1**	**10^6/L2**	**10^7/L1**	**10^7/L2**
C267U	-	-	-	-	+	-	-	-	-	-
C343U	-	-	-	-	-	-	-	+	-	-
U609C	-	-	-	-	+	-	-	-	-	-
A1088G	-	-	+	-	+	+	+	+	+	+
U1295C	-	-	-	-	-	-	-	-	+	-
G1312A	-	-	-	-	-	-	-	+	-	-
G1371A	-	-	-	-	-	-	-	+	-	-
C1649U	-	-	-	-	-	+	+	-	+	+
U1665C	-	-	-	-	-	-	+	-	-	-
A1827G	+	-	-	-	+	-	-	+	-	-
G1835U	-	-	-	-	-	-	-	-	+	-
A1930G	+	+	-	+	-	-	-	-	-	-
U2016C	-	-	-	-	-	+	+	-	+	+
U2135C	-	-	-	-	-	-	-	+	-	-
A2222C	-	-	-	-	+	+	+	+	+	+
C2228U	+	+	+	+	-	-	-	-	-	-
U2766C	-	+	-	-	-	-	-	-	-	-
C3201A	-	-	+	+	-	-	-	-	-	-
U3311G	-	-	-	-	+	+	+	+	+	+
U3317C	-	-	-	-	-	-	+	-	-	-
U3447C	-	-	-	-	+	-	-	-	-	-
U3761C	-	-	-	-	-	-	+	-	-	-
U3784C	+	-	-	-	-	-	-	-	-	-
C3879A	-	+	-	-	-	-	-	-	-	-
C3903U	-	-	-	-	-	-	+	-	-	-
G3945A	-	-	-	-	-	+	+	-	+	+
G3959A	-	-	+	-	-	-	-	-	-	-
U3968C	-	-	-	-	-	-	+	-	-	-
C4025U	+	-	-	-	-	-	-	-	-	-
C4134U	-	-	-	-	+	-	-	-	-	-

[1]The sign – refers to the absence of the indicated mutation and the sign + its presence in the corresponding evolutionary lineage.
