# Supplementary material for: Standing Genetic Diversity and Transmission Bottleneck Size Drive Adaptation in Bacteriophage Qβ
Source: Int J Mol Sci. 2022 Aug 9;23(16):8876. doi: 10.3390/ijms23168876 (PMC9408265; doi:10.3390/ijms23168876)
Supplement: Supplementary file 1 [file ijms-23-08876-s001.zip › Table S3.pdf]

**Table S3.** Sequence of the primers used to prepare single mutants through site-directed mutagenesis.

| **Mutant** | **Primers[1]** |
|---|---|
| $Q\beta_{A2187C}$ | 5'-TCGATAAATGCTTATTGCTCTCTT**C**GCGATATTGCGGCC-3'<br>5'-GGCCGCAATATCGC**G**AAGAGAGCAATAAGCATTTATCGA-3' |
| $Q\beta_{A1088G}$ | 5′-GTCAGCTATATCATAATATCG**G**TATTGTAGACGGCTTTGACAG-3′<br>5′-CTGTCAAAGCCGTCTACAATA**C**CGATATTATGATATAGCTGAC-3′ |
| $Q\beta_{A1930G}$ | 5′-TAATGCTGTTGAACTCC**G**GCCTCGCGAATTTGATG-3′<br>5′-CATCAAATTCGCGAGGC**C**GGAGTTCAACAGCATTA-3′ |
| $Q\beta_{C2228U}$ | 5′- GATGGCGTGATAGTTGG**T**TTTTGGCGCGATCCATC-3′<br>5′- GATGGATCGCGCCAAAA**A**CCAACTATCACGCCATC-3′ |
| $Q\beta_{U3311G}$ | 5′-GTTGTTACCTACGAGAAGAT**G**TCTTCTATGGGTAACGG-3′<br>5′-CCGTTACCCATAGAAGA**C**ATCTTCTCGTAGGTAACAAC-3′ |

[1]The position mutated (with respect to the virus $Q\beta_{WT}$) is indicated in bold.
