# Supplementary material for: Standing Genetic Diversity and Transmission Bottleneck Size Drive Adaptation in Bacteriophage Qβ
Source: Int J Mol Sci. 2022 Aug 9;23(16):8876. doi: 10.3390/ijms23168876 (PMC9408265; doi:10.3390/ijms23168876)
Supplement: Supplementary file 1 [file ijms-23-08876-s001.zip › Table S4.pdf]

Table S4. Values for the parameters *Nt*, N_0, H_0, *N*, and *H* in the amplicons obtained for Qβ-t2 and Qβ-t25.

**Population**	**Amplicon**[1]	***Nt***[2]	N_0[3]	H_0[4]	***N***[5]	***H***[6]
Qβ-t2	1	324304	309124	13688	266858	30
Qβ-t2	2	379763	358986	26127	294855	49
Qβ-t2	3	277476	262456	15078	223334	39
Qβ-t25 Sample 1	1	286318	274466	14710	235849	84
Qβ-t25 Sample 2	2	380690	359406	31345	279452	71
Qβ-t25 Sample 3	3	439189	408717	20728	351353	75

[1]Amplicons corresponding to population Qβ-t2 were obtained in the same sequencing project as the sample 1 of Qβ-t25 (see Figure S1). [2]Total number of paired reads obtained per amplicon. [3]Paired reads per amplicon after bioinformatics processing (see section 4.7 in Materials and Methods). [4]Number of different haplotypes contained in N_0. [5]Number of reads containing haplotypes represented above a frequency of 0.05% in N_0. [6]Number of different haplotypes in *N*.
